# Identification of BGN and THBS2 as metastasis‐specific biomarkers and poor survival key regulators in human colon cancer by integrated analysis

**DOI:** 10.1002/ctm2.973

**Published:** 2022-11-14

**Authors:** Zhicheng He, Jian Lin, Cheng Chen, Yuanzhi Chen, Shuting Yang, Xianghai Cai, YingYing He, Shubai Liu

**Affiliations:** ^1^ State Key Laboratory of Phytochemistry and Plant Resources in West China, Kunming Institute of Botany Chinese Academy of Sciences Kunming China; ^2^ University of Chinese Academy of Sciences Beijing China; ^3^ School of life Science Yunnan University Kunming China; ^4^ School of Chemical Science and Technology Yunnan University Kunming China

**Keywords:** BGN, biomarker, colon cancer, EMT, metastasis, THBS2

## Abstract

**Background:**

Colon cancer is the second leading cause of death worldwide. Exploring key regulators in colon cancer metastatic progression could lead to better outcomes for patients.

**Methods:**

Initially, the transcriptional profiles of 681 colonrectal cancer (CRC) cases were used to discover signature genes that were significantly correlated with colon cancer metastasis. These signature genes were then validated using another independent 210 CRC cases’ transcriptomics and proteomics profiles, and Kaplan–Meier regression analyses were used to screen the key regulators with patients’ survival. Immunohistochemical staining was used to confirm the biomarkers, and transit knockdown was used to explore their implications on colon cancer cells migration and invasion abilities. The impact on the key signalling molecules in epithelial‐mesenchymal transition (EMT) process that drive tumour metastasis was tested using Western blot. The response to clinical standard therapeutic drugs was compared to clinical prognosis of key regulators using an ROC plotter.

**Results:**

Five genes (BGN, THBS2, SPARC, CDH11 and SPP1) were initially identified as potential biomarkers and therapeutic targets of colon cancer metastasis. The most significant signatures associated with colon cancer metastasis were determined to be BGN and THBS2. Furthermore, highly expression of BGN and THBS2 in tumours was linked to a worse survival rate. BGN and THBS2 knockdown significantly reduced colon cancer cells migration and invasion, as well as down‐regulating three EMT‐related proteins (Snail, Vimentin and N‐cadherin), and increasing the proliferation inhibitory effect of 5‐fluorouracil, irinotecan and oxaliplatin treatment.

**Conclusions:**

CRC metastatic progression, EMT phenotypic transition and poor survival time have been linked to BGN and THBS2. They could be utilized as potential diagnostic and therapeutic targets for colon cancer metastatic patients with a better prognosis.

## Graphical abstract

Overview of the integrated analysis to discover hall marker in colon cancer metastasis.

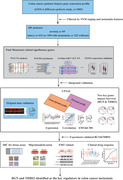

## 1 BACKGROUND

Colorectal cancer (CRC) is the world's second‐deadliest cancer and the third‐most‐commonly diagnosed malignancy.^1^, ^2^, ^3^ Lacking of meaningful biomarkers, CRC is a complex tumour that is difficult to diagnose and treat in the early stage, resulting in the majority of patients being diagnosed in the middle and advanced stages. Furthermore, due to its anatomical location in the body, colon cancer is more difficult to diagnose and treat than rectum cancer. As a result, most patients would only receive radiotherapy and chemotherapy treatment because they missed out on the best surgical treatment option when they were diagnosed, resulting in a poor prognosis and survival rate.^4^, ^5^ Colon cancer metastasis (including lymphatic and distant metastasis) remains a major cause of mortality in newly diagnosed colon cancer patients despite advances in early detection and treatment.^6^ Multiple biological processes are involved in tumour metastasis, which includes abnormal cell cycle control, disruption of the normal cytoskeleton and extracellular matrix, infiltration of adjacent tissues and metastasis to lymph nodes and other organs.^7^, ^8^ The epithelial‐mesenchymal transition (EMT), which occurs when epithelial cells acquire a mesenchymal phenotype,^9^, ^10^, ^11^ is a critical pathogenic event in tumour formation. EMT induces the mesenchymal phenotype, which is associated with migration, invasion, and metastasis.^11^ As a result, it is vital to develop novel metastasis‐specific diagnostic biomarkers or prospective therapeutic targets that might assist forecast outcomes and guide more effective therapy.

Multi‐omics has recently been applied to cancer research, combining large‐scale proteomics and genomics analysis between tumour and normal tissues to provide a more comprehensive understanding of cancer oncogenesis, with datasets generated and deposited in the clinical proteomic tumour analysis consortium (CPTAC).^12^ The cancer consortium proteogenomics profiles offers unique novel approaches to advance understanding the molecular underpinnings of cancer and to aid in the discovery of cancer biomarker.^13^

In this study, a novel combination screening strategy was employed to discover potential novel biomarkers that work as critical regulator in CRC metastasis by integrated clinical multiple omics profile analysis with cellular functions assay. Initially, the gene expression profile of 681 clinical samples from colon cancer was obtained from the gene expression omnibus (GEO) (normal sample 69 cases, 322 non‐metastatic cases and 290 metastatic cases) and The Cancer Genome Atlas (TCGA). The significant correlation between CRC expression profile and metastasis pathological characteristics pathological feature was performed by weighted gene co‐expression network analysis (WGCNA) to identify the most significant signature genes associated with colon cancer metastasis, which is widely used not only for the discovery of new biomarkers for disease diagnose, but also for the search potential drug targets using a systems biology algorithm.^14^, ^15^ The identified signature genes were validated through another independent clinical proteogenomics profiles of CRC clinical cases (*n* = 86) in the CPTAC database. Finally, the impact of these potential hallmark genes on colorectal cancer metastatic progression, EMT phenotypic transformation, cellular functions and short survival time has been investigated. This study may be helpful to develop new diagnostic biomarkers and therapeutic targets for colon cancer metastases diagnosis and therapy strategy.

## 2 METHODS

### 2.1 Patients’ expression profile dataset collection

**FIGURE 1:**
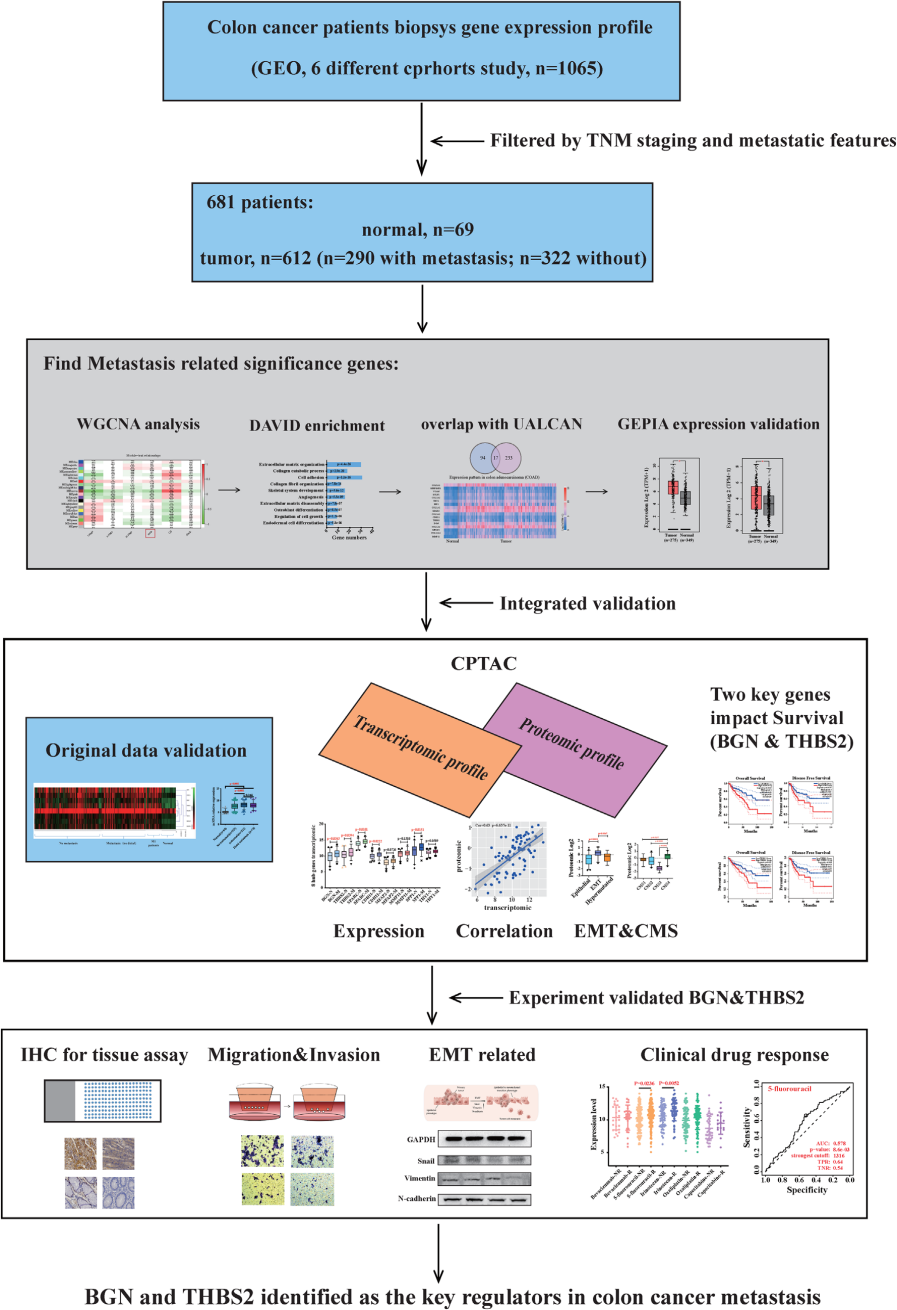
Overview workflow of the data analysis and experimental design

This study's work flow was well detailed (Figure 1). The GEO database (http://www.ncbi.nlm.nih.gov/geo/) was used to obtain expression profiles of CRC patients. The GPL570 (HG‐U133_Plus_2) Affymetrix Human Genome U133 Plus 2.0 Array was used to create probe annotations and platform medidata (Affymetrix, Santa Clara, CA, United States). For the first identification and validation of prognostic biomarkers in metastasis colon cancer, six cohorts of CRC patients’ expression profiles (**GSE9348, GSE8671, GSE47908, GSE62932, GSE29623 and GSE40967**) were employed. In the initial research, 681 patients’ profiles were used, including 69 normal colon samples and 612 colon cancer samples, based on TNM staging and metastatic features (322 without metastasis and 290 metastasis samples).

### 2.2 Data processing and analysis

The datasets contained about 22 000 target genes, which contains total of 45 000 probes that correspond to measuring changes in gene expression profiles that are correlated with the physiologic profile of colon cancer tissues. Each gene expression value was normalized and transformed using log2. Among all characteristics, metastatic feature was used as the focus of our investigation. In these features, biopsy samples were divided into four subgroups, including normal colon tissues as control (*n* = 69) and colon cancer tissues (without metastasis, *n* = 322, with metastasis but no distal metastasis, *n* = 212 and with distal metastasis patients, *n* = 78). The quality of microarray was evaluated using sample clustering based on the distance between different samples in Pearson's correlation matrices, and a height cut of 200 was chosen to identify potential microarray outliers. Four samples (GSM972311/972046/972213/972021) of colon cancer tissues were detected as outliers and ignored in the subsequent analysis.

### 2.3 Bioinformatic analysis process

The bioinformatic analysis involved the following processes: (1) Based on “R project” (version: R 3.6.1) and WGCNA code, the obtained 681 samples were clustered to construct a cluster tree, and the correlation co‐efficient among samples of each gene expression profile was further calculated. According to the threshold value (0.85), the data lower than the threshold value could be removed. (2) According to the connectivity pattern of the genes expression level, these genes were divided into different modules (a group of genes with a similar expression profile, which always has similar expression changes in a physiological process or in different tissues). The correlation between different modules and the studied phenotype was calculated, the module with the strongest correlation with the studied disease characteristics were selected, and the correlation between modules was further verified by matrix and tree diagram. (3) One hundred eighty‐two genes with the strongest significance and correlation were extracted from the significance modules. David database was used to enrich the signalling pathway, cell components and biological function of selected genes, and there are 111 genes corresponding to each significant enrichment were selected for further analysis. (4) The selected genes overlapped with top 250 up‐regulated genes in colon adenocarcinoma (COAD) of UALCAN database, and 17 genes whose gene expression was up‐regulated in colon cancer were identified as candidate genes. (5) Gene expression profiling interactive analysis (GEPIA) (http://gepia.cancer‐pku.cn/) was explored to compare the mRNA levels of these candidate genes in 275 colon cancer tissues and 349 normal tissues (this expression data derived from TCGA‐COAD). The top 250 up‐regulated genes in COAD, which were featured in UALCAN database, were filtered with selected 111 genes, and overlapping genes that increased in colon cancer were identified as prospective signature genes. Signature genes are genes that have a significant difference in expression between colon cancer and normal tissue. MeV 4.9.0 (Multiple ExperimentViewer) was used to analyse and present the expression pattern of signature genes in normal samples, cancer patients and metastatic cancer patients as heat‐map, and GraphPad Prism 6.0 (GraphPad Software, La Jolla, CA, USA) was used to analyse differences in expression level analysis. The individual gene expression in each group was represented as means ± standard error of the mean (SEM), which represented the group's cases distribution. The fold change ratio was employed to quantify the expression level comparison.

### 2.4 Signature genes validation through proteogenomics profiles of CRC patients

The CPTAC data portal provided independent proteogenomics profiles of CRC patients^12^ (86 cases of colon cancer patients: 49 without metastasis, 37 with metastasis), which were utilized to validate signature genes using the python cptac package.^13^ In addition, differences in signature genes’ transcription and protein expression levels between non‐metastatic and metastatic cases were investigated, respectively. The correlation between the proteomics and transcriptomics level was also investigated. Furthermore, the expression of signature genes in cancer tissues from patients with epithelial, EMT and hypermutated phenotypes was compared. Finally, the protein expression pattern of eight signatures was analysed in colon cancer patients’ proteomic dataset, which was used to sort colon cancer patients in CRC consensus molecular subtypes.^16^, ^17^

### 2.5 Cell culture and plasmid transfection

The impact of BGN, THBS2 and MFAP2 on cellular function in vitro was investigated using CRC cell lines HT29 and HCT116. The GeneChem company created and constructed short‐hairpin RNA (shRNA) targeting BGN (shBGN), THBS2 (shTHBS2) and control shRNA (mock) (Shanghai, China, Table S13). Transfections were carried out according to the Lipofectamine 2000 (Invitrogen) protocol. The knockdown of BGN in HT29 and THBS2 in HCT116 cells were confirmed by Western Blotting analysis after 2 weeks of screening in media with 0.8 μg/ml puromycin (Sigma‐Aldrich Co.). In cell proliferation, colony formation and transwell assays were validated in these stable transfected cell lines.

### 2.6 Statistical analysis

Data were presented as means ± SEM. Significance of differences for the values were determined using the student *t*‐test with Prism software (GraphPad Software, Inc. San Diego, CA). A significant difference was defined as a *p*‐value less than .05.

Detailed descriptions of the others experimental methods were described in the section of Supplementary Materials and Methods.

## 3 RESULTS

### 3.1 Identify the module and genes significant related to CRC metastasis

**FIGURE 2:**
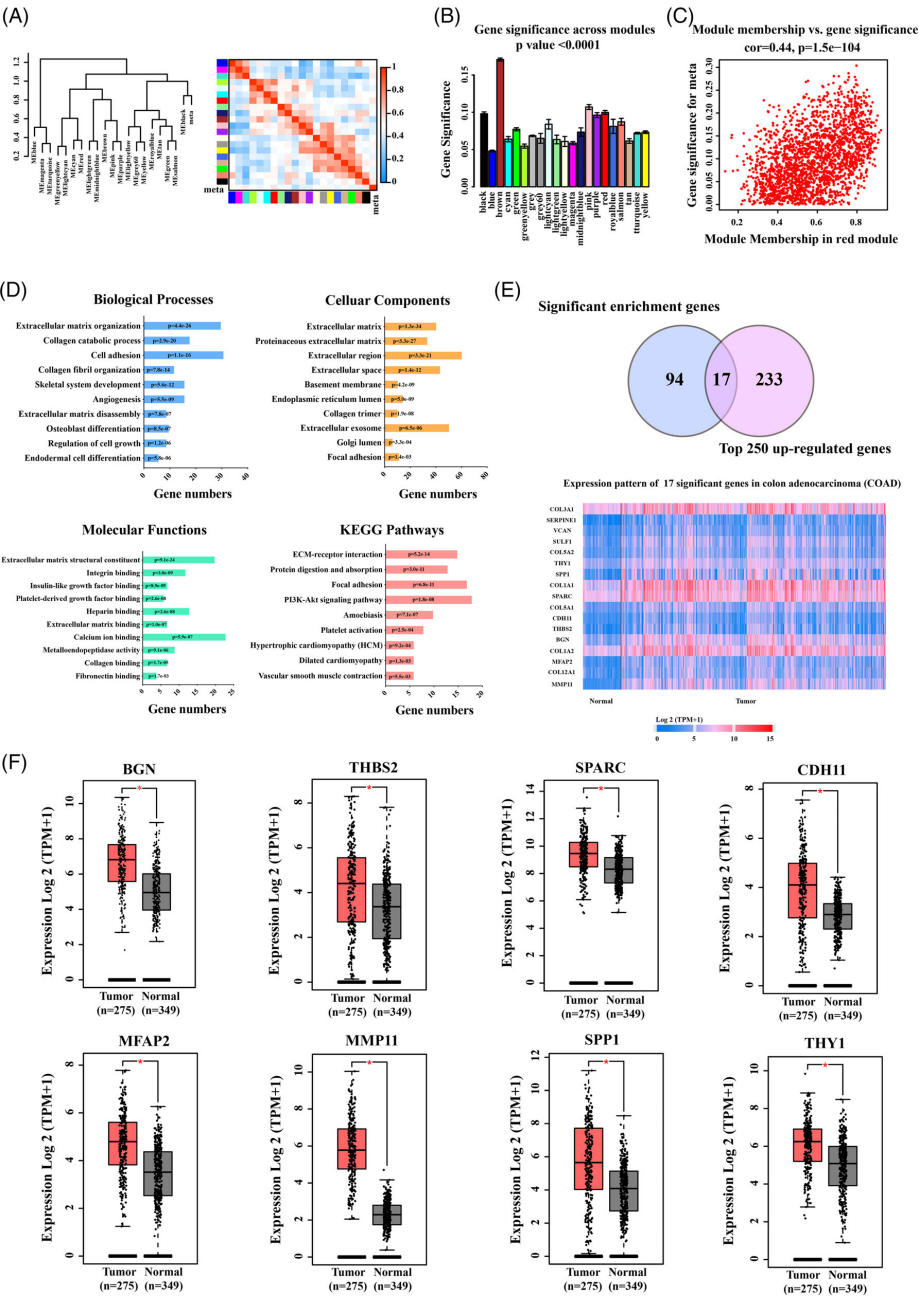
Identified the signature genes significantly associated with colon cancer metastasis. Clustering dendrogram of the eigengenes and adjacency with cancer metastasis dissimilarity based on topological overlap. (A) The left panel shows a hierarchical clustering dendrogram of the eigengenes in which the dissimilarity of eigengenes (EI, EJ is given by 1 – cor (EI, EJ)). The heatmap in right panel shows the eigengene adjacency (AI J = (1 + cor (EI, EJ))/2). The Gene Significance of all modules identified correlated with colon cancer metastasis. (B) Distribution of average gene significance and errors in the significant modules associated with cancer metastasis. There is a highly significant correlation between genes significance (GS) and module membership (MM) in this module that the most important elements of module tend to be highly correlated with colon cancer metastasis. (C) Comprehensive analysis showed that red module that the most was the most significantly with colon cancer metastasis. (D) The top 10 functional and pathway enrichment analysis of the significant genes in red module that associated with colon cancer metastasis. Significantly biological processes, cellular components, molecular functions and Kyoto Encyclopedia of Genes and Genomes (KEGG) pathway enrichment analysis by DAVID (*p* < .01). (E) The 17 genes as candidate of signature genes were identified by overlapped 111 genes by DAVID enrichment analysis and top250 up‐regulated genes in COAD by UALCAN database. (F) The eight signature genes were filtered by GEPIA database through 17 genes candidates, which are significantly over‐expression in colon cancer patients. The data were expressed as the means ± standard error of the mean (SEM) (*p value < .001)

The gene expression profile data of the tumour metastasis features were clustered and divided into 21 modules (Figure S1D). The correlation significance of module and pathological features was determined using module significance (MS) correlation and statistics *p*‐value. Turquoise module (*t*‐value = 0.11, *p*‐value =.003), red module (*t*‐value = 0.16, *p*‐value = 3e‐05) and black module (*t*‐value = 0.15, *p*‐value = 6e‐05) were all significantly positive correlated to colon cancer metastasis pathological feature (Figure S1D). By calculating and comparing the module memberships (MMs) correlation and genes significance (GS), the significant modules that correlated to the tumour metastasis feature were determined. Turquoise module (cor = 0.34, *p*‐value = 2.1e‐173), red module (cor = 0.44, *p*‐value = 1.5e‐104) and black module (cor = 0.29, *p*‐value = 4.3e‐41) were identified as significant modules that associated with tumour metastasis when combined with the Eigengene dendrogram analysis and the module‐trait relationship correlation results (Figure S2A–C). Although the black module was tightly clustered with tumour metastasis feature, red module had the strongest correlation with tumour metastasis status (Figure 2C, Figure 2A,B, Table S2), which contained 1457 genes (Table S2). Each significant module's MM scatter plot was plotted against its GS, with each point representing a gene contained in a module (Figure S2).

### 3.2 Functions and pathways enrichment analysis and signature genes screening

In the red module, 191 genes with higher significance (screening standard: GS > 0.18, MM > 0.33) were chosen, filtered in Genclip (http://ci.smu.edu.cn) and DAVID enrichment analysis was used to retrieve 181 genes (Table S3). These 181 significance genes were investigated biological functions by gene ontology enrichment and Kyoto Encyclopedia of Genes and Genomes pathway analyses. The top 10 enrichment signalling pathways were summarized, which are enriched in the physiological processes and signalling pathways related to the extracellular matrix, such as angiogenesis and cell growth regulation (Figure 2D), both of which are involved in colon cancer metastasis. Furthermore, the significance genes were narrowed down to 111 genes (Table S4), and 17 genes were identified as signature genes candidate utilizing over‐expressed filter by blasting through the top 250 up‐regulated genes in COAD using the UALCAN database (Table S5, Figure 2E). Finally, the clinical cases from GEPIA database (TCGA‐COAD) were used to evaluate the expression levels of candidate genes in tumour and normal samples. Eight significantly over‐expression genes in tumour tissues (BGN, THBS2, SPARC, CDH11, MFAP2, MMP11, SPP1 and THY1) were defined as significantly signature genes that implicated in colon cancer metastasis progress (Figure 2F).

### 3.3 Expression pattern of signature genes

**FIGURE 3:**
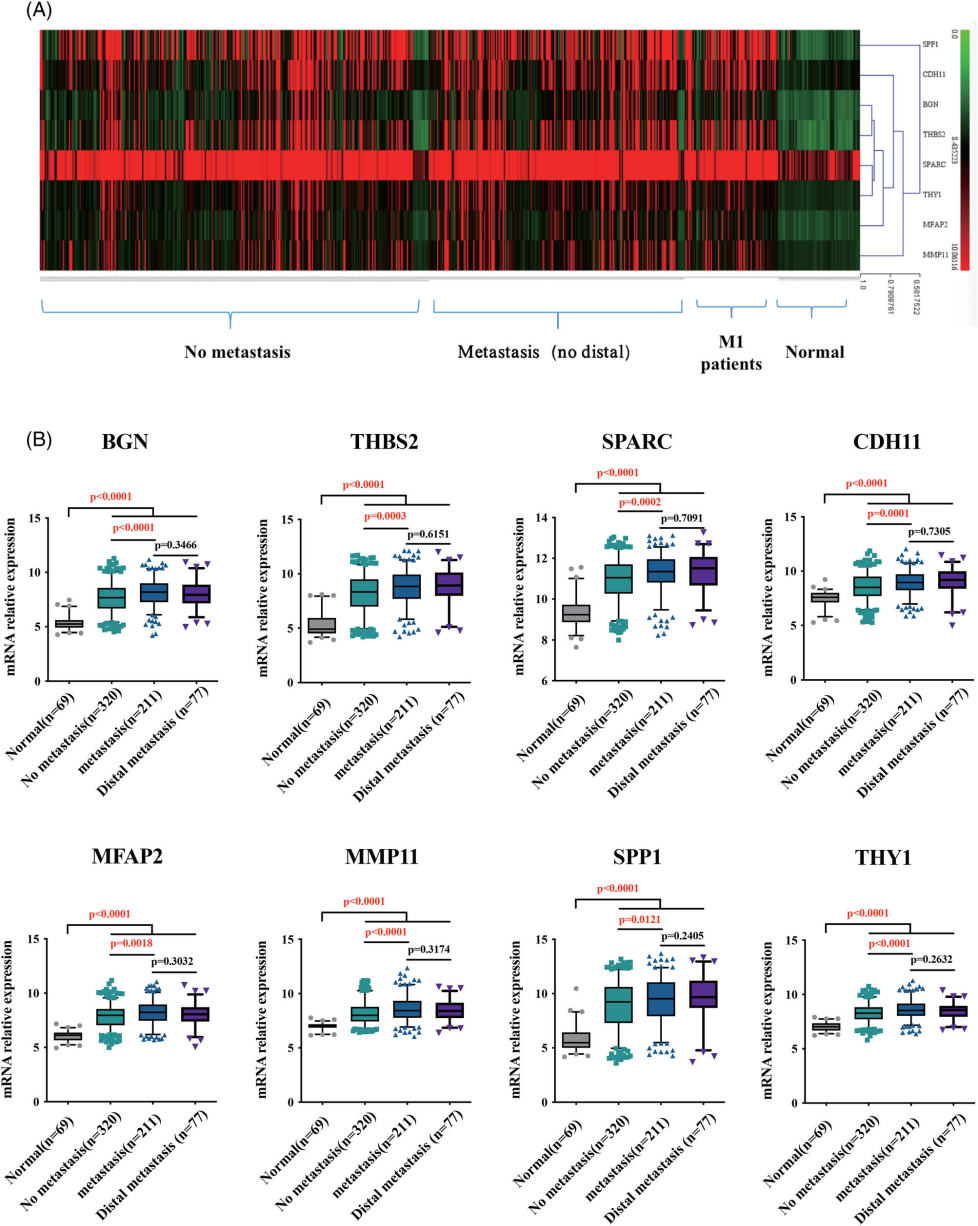
The expression pattern of eight signature genes in normal, without and with metastatic colon cancer samples analysed by original gene expression profile data sets. (A) Heat‐map. (B) In original data sets analysis, eight hub genes expression levels in metastasis and distal metastasis samples are significantly higher than in normal and no‐metastasis samples

According to the pathological feature, the original gene expression profiles data were divided into four groups: normal, non‐metastatic, metastatic and distal metastatic, and explore the association between eight signature genes and colon cancer metastasis. A heat‐map was created to represent the expression pattern of eight signature genes in each group's (Figure 3A). In three tumour groups, eight signature genes exhibited significantly higher expression level than in the normal group (*p* < .0001) (Figure 3B). Furthermore, the expression level of metastatic group was significantly higher than that of the non‐metastatic group in tumour groups (*p* < .001), while there was no significant difference between the metastatic and distant metastatic groups (*p* > .05).

### 3.4 Correlation of signature genes in transcriptomic and proteomic profiles

**FIGURE 4:**
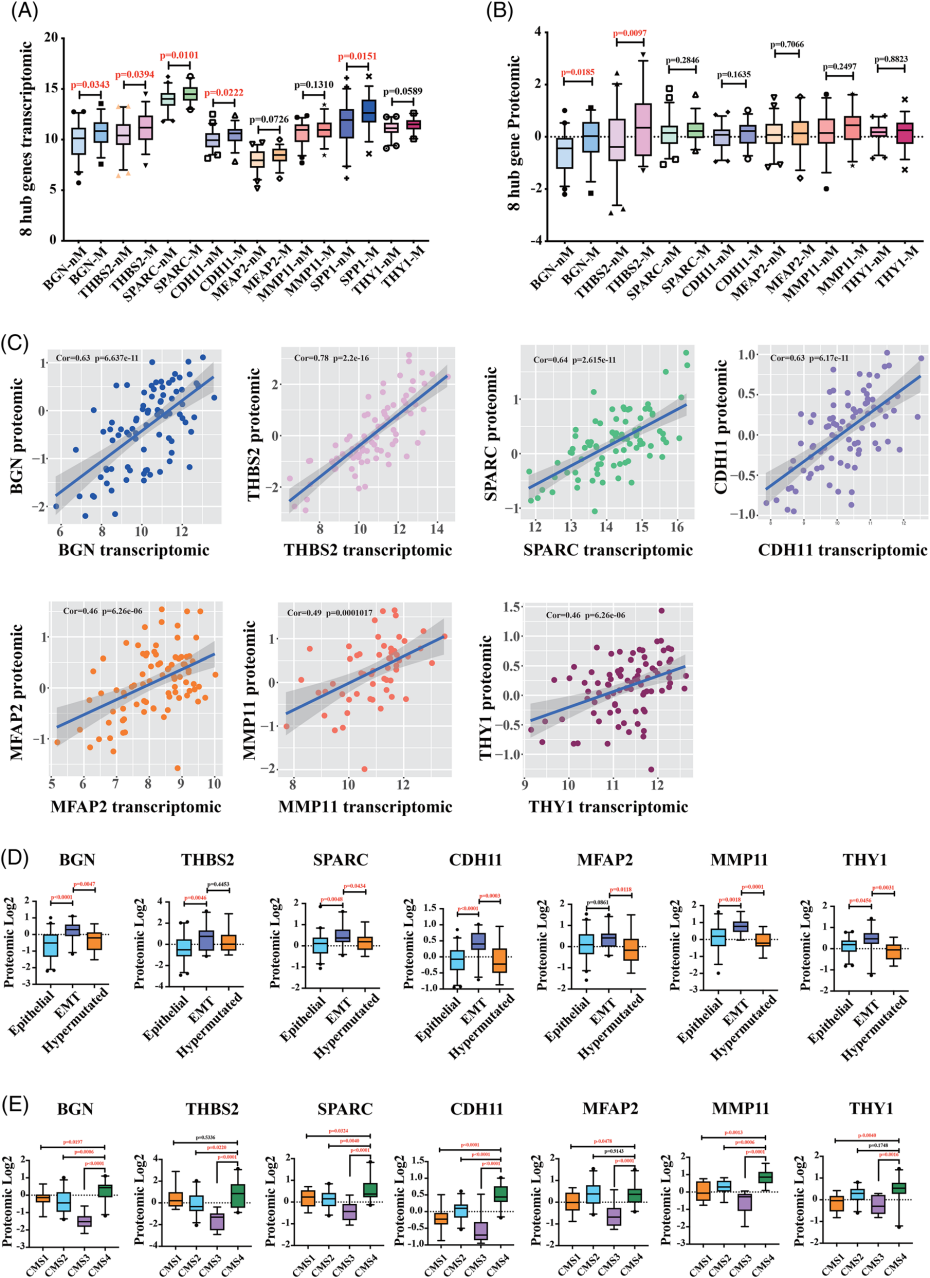
Transcriptomic and proteomic expression pattern of eight signature genes analysis from CPTAC. (A) In CPTAC (The National Cancer Institute's Clinical Proteomic Tumour Analysis Consortium) transcriptomic data analysis, eight hub genes transcription levels in metastasis samples are significantly higher than no‐metastasis samples. (B) CPTAC proteomic data analysis indicated that protein expression level of BGN and THBS2 in metastasis samples are significantly higher than no‐metastasis samples (nM: no metastasis; M: metastasis). (C) Correlation analysis revealed a significant positive correlation between the proteomic and transcriptomic of 7 genes. (D) Protein expression level of seven signature genes in patients with epithelial, epithelial‐mesenchymal transition (EMT) and hypermutated phenotype. (E) Protein expression level of seven signature genes in patients from four different consensus molecular subtypes (CMS1∼4)

The independent transcriptomic and proteomic data of CRC from CPTAC for further evaluation of these eight signature genes. There are five genes (BGN, THBS2, SPARC, CDH11 and SPP1) in metastatic cancer tissues that are significantly higher than non‐metastatic cancer tissue at the transcriptomics level (Figure 4A), while only BGN and THBS2 were significant difference in protein expression between metastatic and non‐metastatic cases (Figure 4B). Furthermore, the transcription and protein expression level of seven signature genes were not only significantly positively correlated in tumour samples (Figure 4C), but also significantly positively correlated in metastasis and non‐metastasis tumour samples (Figure S3A–G). However, their protein expression levels were significantly higher in cancer tissues than in normal tissues. Interestingly, the protein expression levels of two signature genes (THBS2, SPARC, MFAP2, MMP11 and THY1) were significant lower in normal tissues (Epithelial) than those in non‐metastatic and metastatic patients’ cancer tissues (Figure 4D), although there was no significant difference in protein expression levels between non‐metastatic and metastatic patients. Furthermore, EMT tumour tissues had significantly higher transcription expression of seven signature genes than epithelial and hypermutated tumour tissues. Protein expression in EMT tumour tissues is also significantly higher than in epithelial or hypermutated phenotype (Figure 4D). These results suggested that these signature genes could be important regulators in colon cancer metastasis progression.

Colon cancer is a heterogeneous disease^18^ and divided into four subtypes by the current authority classification system (CMS1∼4).^19^, ^20^ EMT is associated with tumour metastasis and is the important characteristic of the CMS4 patients.^19, 20^ Therefore, the protein expression of seven signature genes in the context of four CMS1∼4 subtypes was used to investigate the relationship between signature genes and EMT. The proteomic expression levels of signature genes (BGN [0.2787], SPARC [0.5775], CDH11 [0.4884] and MMP11 [0.8907]) were specifically higher in the CMS4 patients versus other subtypes (CMS1∼3) (Figure 4E). The THBS2 for distinguishing CMS4 versus CMS2–3 subtypes in CRC were 0.7528, and MFAP2 and THY1 for CMS4 versus CMS1/3 subtypes were 0.3491 and 0.4303, respectively (Figure 4E). With higher expression level in EMT state, these genes may be through EMT process to involve in the process of prominent transforming growth factor β activation, stromal invasion and angiogenesis, and are correlated with colon cancer metastasis.^19^, ^20^ These results suggested that these signature genes may influence the metastasis of colon cancer by affecting epithelial‐to‐mesenchymal transition processes and involve in the process of tumour metastasis.

### 3.5 Identified dominant hallmark genes correlated with poor survival in CRC patients with metastasis

**FIGURE 5:**
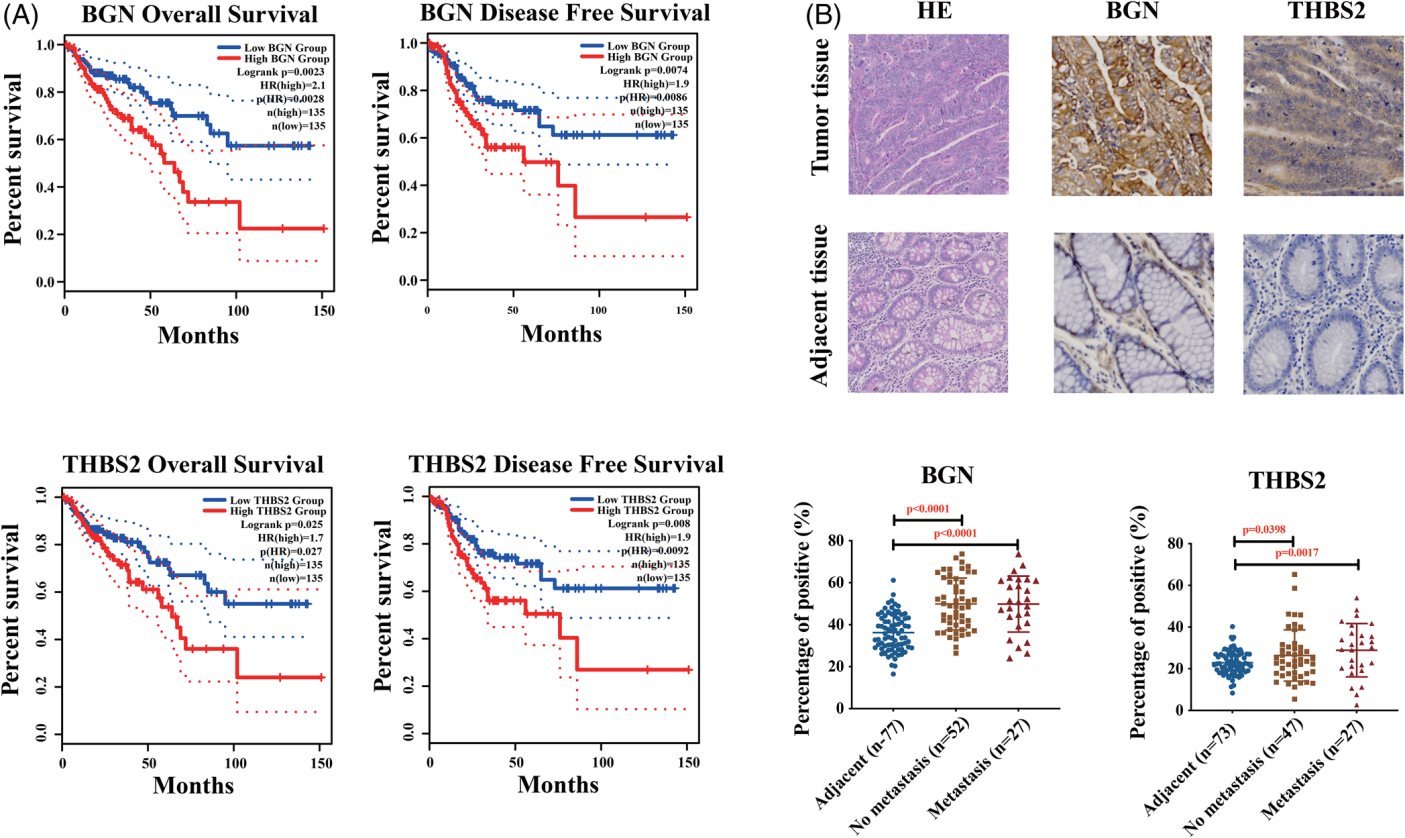
Dominant hallmark genes correlated with poor survival in colon cancer metastasis patients. (A) Overall survival and disease‐free survival analysis of BGN and THBS2. (B) Immunohistochemical analysis of protein expression level of BGN and THBS2 in colon cancer tissue assays

Colon cancer patients with higher expression of BGN and THBS2 were associated with a significantly lower survival time by exploring the GEPIA database^21^ (Figure 5A and Figure S4A–F). Therefore, these two genes were chosen as representative hallmark genes and experimented on. IHC staining results indicated that BGN and THBS2 were significantly up‐regulated in most of the colon cancer tissues than health adjacent tissues (Figure 5B). Furthermore, cancer tissues were classified as non‐metastatic and metastatic depending on whether the individuals had lymph node metastases. The expression of two genes was analysed in the two groups, and there was no significant difference in expression (*p* > .05) (Figure 5B). It could be caused by few metastatic samples to validate the variations in these genes expression between metastatic and non‐metastatic groups, and the statistical results do not accurately reflect the differences. It is suggested that these hallmark genes may play important roles in tumour genesis, progression and metastasis.

### 3.6 Knockdown BGN and THBS2 impaired the proliferation, migration and invasion in colon cancer cells

**FIGURE 6:**
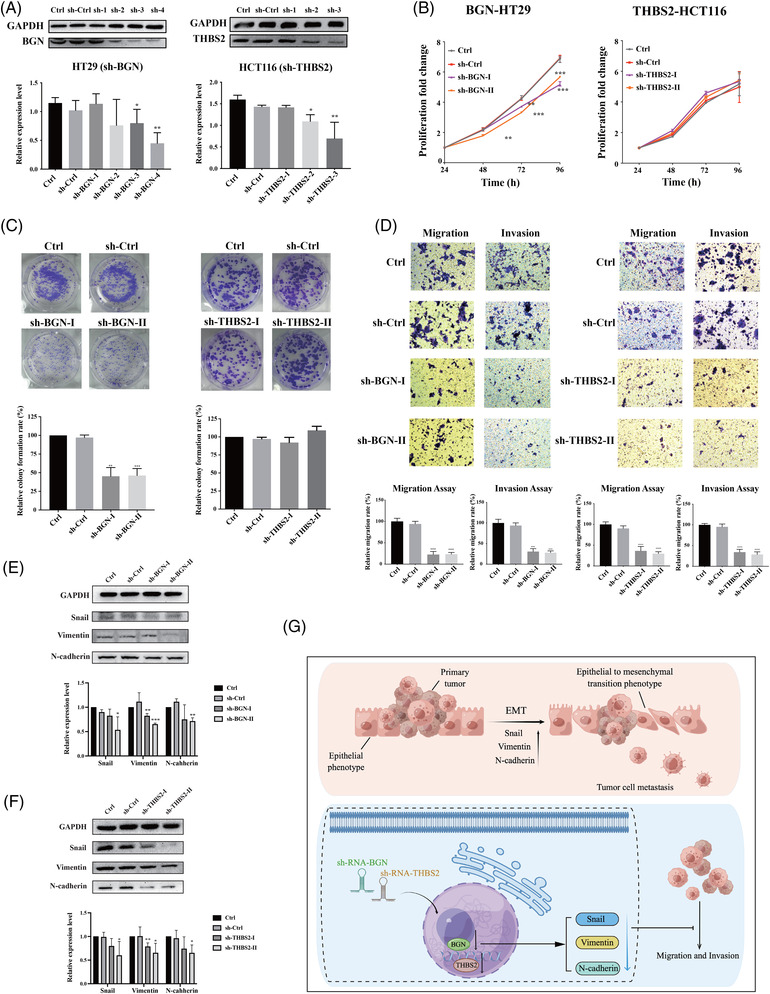
Experimental validation of representative biomarkers. (A) Western blot confirmed BGN and THBS2 stable knockdown in colon cancer cells constructed by plasmids containing BGN and THBS2‐targeting shRNA. (B) CCK8 assay showed that knockdown of the expression levels of BGN reduced the proliferation ability of colon cancer cell lines. (C) Colony formation assay showed that knockdown of the expression levels of BGN reduced the ability of colony formation of colon cancer cell lines. (D) Differences in migration and invasion ability of cells after knockdown of BGN and THBS2 expression levels, and the migration and invasion ability of the cells were both decreased when these two genes were knocked down. (E and F) Three proteins during epithelial‐mesenchymal transition (EMT) (Snail, Vimentin and N‐cadherin) were decreased by knockdown the expression of BGN and THBS2. (G) BGN and THBS2 may inhibit colon cancer cell migration and invasion by participating in the regulation of EMT process

It is thought that BGN and THBS2 overexpression contributes to the abnormality of colon cancer cells. The lentivirus shRNA constructs successfully generated scramble control and Hallmark genes (BGN, THBS2) knockdown in HT29 and HCT116 cells, respectively. To avoid off‐target effects, each target gene was initially screened with three or four different shRNA constructs, and then selected two independent clones for the next step functional assay, which were validated by Western blot with clearly knockdown (Figure 6A). The ability of colon cancer cells in proliferation, invasion and migration were evaluated, and BGN protein knockdown significantly reduced HT29 cells proliferation (Figure 6B), clone formation numbers in vitro (Figure 6C), migration and invasion (Figure 6D) as measured by the transwell assays, while THBS2 knockdown has little influence on colon cancer cells proliferation or colony formation abilities and only significantly reduced HCT116 migration and invasion ability. It is suggested that BGN and THBS2 were critical in promoting colon cancer cells proliferation and tumourigenicity, and influent the migration and invasion of colon cancer cells during cancer metastasis. Furthermore, these key regulatory molecules in colon cancer metastasis and EMT progression, Snail, Vimentin and N‐Cadherin were significantly down‐regulated in the (BGN, THBS2) knockdown colon cancer cells (Figure 6E–F), which are prominent inducer of EMT, strongly repress E‐cadherin expression and promote metastasis.^22^, ^23^, ^24^ It is strongly suggested that decreased expression of these genes (BGN, THBS2) could block the dominant signalling molecules in colon cancer metastasis. It is also proposed the potential action model of BGN and THBS2 regulating EMT and metastasis processes in colon cancer cells (Figure 6G).

### 3.7 Hallmark genes expression correlated with clinical drug treatment

**FIGURE 7:**
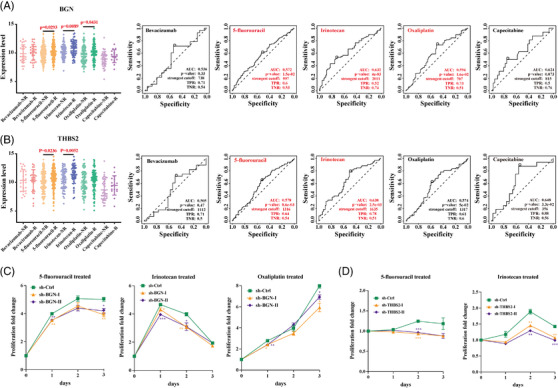
Correlation between five clinical drugs treated response and two key biomarkers expression levels in colon cancer. (A) Patients who responded to 5‐fluorouracil, irinotecan and oxaliplatin had significantly higher levels of BGN expression than those who did not responded. AUC and *p* value of ROC of BGN high expression level in treatment with five drugs and 5‐fluorouracil, irinotecan and oxaliplatin have well efficacy. (B) Patients who responded to 5‐fluorouracil and irinotecan had significantly higher levels of THBS2 expression than those who did not responded. AUC and *p* value of ROC of THBS2 high expression level in treatment with five drugs and 5‐fluorouracil and irinotecan have well efficacy. (C) Effects on proliferation of stably transfected sh‐BGN cell lines after treated with 5‐fluorouracil, irinotecan and oxaliplatin. (D) Effects on proliferation of stably transfected sh‐THBS2 cell lines after treated with 5‐fluorouracil and irinotecan

The fluorinated analog of uracil, 5‐fluorouracil (5‐FU)‐based therapies,^25^ including FOLFOX (5‐FU, oxaliplatin) or FOLFIRI (5‐FU, irinotecan), Bevacizumab,^26^ metastasis target drug, Capecitabine,^27^ the maintenance therapy drug, have been used as the standard therapy for advanced CRC or metastatic CRC in clinical operation. To further understand the role of these hallmark genes in clinical medication and evaluate their potential application in clinical diagnosis and treatment, it was evaluated that the correlation between the hallmark gene expression and the 5‐FU‐based treatment response and clinical outcome in patients. Patients who reacted to treatment of 5‐fluorouracil, irinotecan and oxaliplatin had significantly higher BGN expression levels than those who did not (Figure 7A), while patients who responded to treatment of 5‐fluorouracil and irinotecan had significantly higher levels of THBS2 than those who did not (Figure 7B). Furthermore, the treatment of 5‐fluorouracil and oxaliplatin had significantly reduced proliferation of BGN knockdown HT29 cells (Figure 7C) and THBS2 knockdown HCT116 cells than control cells (Figure 7D), respectively. It is suggested that these hallmark genes (BGN, THBS2) may not only be novel EMT indicators but also provide direction for several clinical medications utilized in colon cancer treatment. Further research into the molecular mechanism of these genes’ roles in the process of EMT may aid in elucidating the mechanism of colon cancer metastasis and provide a foundation for developing new methods for treating and inhibiting colon cancer metastasis.

## 4 DISCUSSION

In this study, integrated clinical CRC multiple omics profile analysis and cellular functions assays, a combined strategy was employed to discover the potential novel biomarkers that work as key regulators in CRC metastasis. Initially, the whole transcription profiles of colon cancer patient's samples were screened and filtered to identify module that the most correlated with colon cancer metastasis by WGCNA. The potential eight signature genes were filtered by COAD and GEPIA database, and blast with proteogenomics profiles of 210 clinical samples in CPTAC,^12^ which offers unique novel approaches to analyse global proteomic differences between tumour and normal tissues and explore the correlation in transcriptomic and protein levels and validate the reliability of biomarkers. BGN and THBS2 were identified to be key regulators of CRC metastatic progression, EMT phenotypic transition and with poor survival time. Furthermore, the hall markers of BGN and THBS2 were intensively validated findings for key regulators candidate of colon cancer metastasis using knockdown and cellular functions assay and evaluated the synergic effect of treatment combined with 5‐Fluorouracil (5‐FU) first‐line clinical drugs.

EMT is important for development, and the processes that underpin it are reactivated in wound healing, fibrosis and cancer progression.^9^, ^10^, ^28^ It also causes the mesenchymal phenotype in tumour tissues, which is linked to tumour cells migration, invasion and metastasis, as well as treatment resistance.^11^ BGN is a member of the small leucine‐rich proteoglycans family, which encodes a glycoprotein core that has been modified.^29^ BGN was the first detected in bone tissue^30^ and has been linked to overexpression in a number of malignancies, including gastric cancer,^31^ esophageal squamous cell carcinoma^32^ and colon tumours.^33^ BGN plays a role in tumour growth, adhesion and invasion.^34^, ^35^, ^36^, ^37^ BGN is a component of the ECM that helps to scaffold collagen fibrils and regulates cell signalling,^38^ and it simulates EMT in a variety of cancers,^39^ and it is regarded as a novel mesenchymal marker for the EMT,^40^ and it probably contributes to malignant cancer cells gaining migratory and invasive abilities,^41^ and it is regulated by the transforming growth factor‐beta signalling pathway, which is a key regulator of the EMT process.^42^ BGN has also been discovered as a progressive biomarker in the transition from normal colon mucosa to adenoma and adenocarcinoma in colon cancer,^33^ and it has been postulated as a potential prognosis biomarker for colon cancer^43^ and has been shown to regulate CRC cells functions.^29^ BGN is thought to play a crucial role in the development of colon cancer. However, the role of BGN in colon cancer metastasis is less well investigated. Our results may reveal the positive significantly correlation BGN and CRC metastasis and validated with large size of clinical patients’ samples.

Thrombospondins (THBS2) is a multifunctional glycoprotein that mediates cell‐to‐cell and cell‐to‐matrix interactions and has a variety of biological effects including angiogenesis, cell motility, apoptosis and cytoskeletal organization. It interacts with MMPs and matrix serine proteases like plasminogen activator to limit tumour growth and angiogenesis in a variety of malignancies, and it is recognized as a key ECM‐modifying enzyme.^51,52^ THBS2 has been identified as a potential biomarker with clinical prognosis in CRC,^44^, ^45^ and the patients with positive THBS2 expression had a significantly lower likelihood of liver metastasis,^46^ and up‐regulated was linked to poor overall survival.^47^ THBS2 promotes CRC cells migration and invasion by regulating the Wnt/beta‐catenin signalling pathway and enhances aerobic glycolysis by regulating HIF‐1a^47^ and thus contributes to CRC progression.^48^ THBS2 expression was significantly higher in cancerous mucosa than in non‐cancerous mucosa, and it was thought to be an angiostatic factor.^49^ THBS2 possesses a binding domain for miR‐203a‐3p in its 3′‐untranslated region, which inhibits CRC progression and metastasis by down‐regulating THBS2 expression.^50^ Lower THBS2 expression was linked to advanced disease status and slower tumour regression after preoperative neoadjuvant concurrent chemoradiotherapy, THBS2 acted as an independent negative prognostic factor in rectal cancer.^51^ In metastatic CRC patients after treated with bevacizumab, patients with higher THBS‐2 stromal expression had a nonsignificant improvement in survival.^52^ Compared with these results, our study revealed that THBS2 had the potential to be used as prognostic biomarker for colon cancer metastasis and clinical treatment.

5‐FU and fluorinated analog of uracil, including oxaliplatin and irinotecan, are used as the first‐line treatment for CRC.^25^ However, the majority of 5‐FU treated patients did not completely destroy tumour cells, induced CRC stem cells activation via the WNT/β‐catenin signalling pathway, and ultimately resulted in recurrence with poor outcomes.^53^ Meanwhile, BGN is overexpressed in colon cancer stem cells and activates NF‐κB pathway, which leads to chemotherapy resistance,^54^ and BGN knockdown enhanced the HT‐29 cells' sensitivity to 5‐FU treatment.^55^ THBS2 promotes CRC cells migration and invasion by regulating the Wnt/beta‐catenin signalling pathway.^47^ In our result, knockdown BGN or THBS2 significantly increased the proliferation inhibitory effect on CRC cells induced by 5‐FU and oxaliplatin treatment. Thus, to improve the poor outcomes of CRC patients with metastasis, it may be therapeutically useful to consider ways suppress THBS2 or BGN expression in CRC and combine this with 5‐FU treatment as a combination treatment strategy.

The advance results of this study share some consistence to previously research studies. BGN and THBS2 had been studied in CRC and played an important role in the progression of CRC metastasis.^45^, ^56^ Previously these results of studies also demonstrate that our data analysis methods have good feasibility and accuracy, which can be used for the discovery of various diseases prognostic indicators and potential therapeutic targets. Combined with our analysis results, it can be inferred that these seven genes may play prognostic roles in metastatic colon cancer and could be used as potential targets to study the mechanism of metastasis of colon cancer.

There are some limitations in this study. Firstly, it was tracking these samples’ pathological features with expression profiles and validate these potential biomarkers against the original patient's pathological feature. The hall mark genes should be tested on the derived cancer cells from patient biopsy. Second, the key signalling pathways regulated by BGN and THBS2 that involved in CRC metastasis were not deeply investigated further due to limitation. Although these genes have been validated as being significantly associated with colon cancer metastasis in cellular function assays, more solid research works are needed to explore the possible regulatory mechanism. In the future, it is necessary and logical to investigate the function and mechanism of these potential signature genes (BGN and THBS2) for colon cancer metastasis using an experimental animal model and patients derived tumour cells.

In summary, this study initially identified five hallmark biomarkers (BGN, THBS2, SPARC, CDH11 and SPP1) as prognostic markers and potential therapeutic targets and validated two representative genes by several experiments. It will provide a basis for further study on their mechanisms in EMT and metastatic colon cancer. BGN and THBS2 may provide guidance for the clinical use of 5‐fluorouracil, irinotecan and oxaliplatin. Our work enriched the prognostic markers associated with metastatic colon cancer and laid foundation for further study the mechanism in colon cancer metastasis. It is suggested that BGN and THBS2 could play a potentially critical role of in the pathogenesis and progression of CRC metastasis. With the deeper progress of researches, BGN and THBS2 might become as a potential biomarker or identified as potential clinical therapy targets for CRC patients with better outcome.

## CONFLICT OF INTEREST

The authors declare that they have no competing interests.

## Supporting information

Figure S1. The sample dendrogram and trait heatmap (A).
Figure S2. The scatterplots of gene significance (GS) for histology vs. module membership (MM) in the black (A) and turquoise (B) modules.
Figure S3. Correlation analysis revealed the significant positive correlation of 7 signature genes between the proteomic and transcriptomic profiles
Figure S4. The overall and DFS survival of patients with different expression level of SPARC (A), CDH11 (B), MFAP2 (C), MMP11 (D), SPP1 (E) and THY1.

Table S1. The biopsy samples information of gene expression profile.

Table S2. All genes in red module.

Table S3. The significant genes related to colorectal cancer metastasis.

Table S4. 111 significant genes filtered by DAVID Enrichment.

Table S5. Top 250 genes up‐regulated in COAD by GEPIA analysis.

Table S6. Transcriptomic profiles of eight signature genes in CAPTC.

Table S7. Proteomics profiles of 7 signature genes.

Table S8. UMS proteomic profiles of 7 signature genes.

Table S9. EMT proteomic profiles of 7 signature genes.

Table S10. CMS1‐4 proteomic profiles of seven signature genes.

Table S11. Five clinical drug treatment response with or without BGN.

Table S12. Five clinical drug treatment response with or without THBS2.

Table S13. shrna constructions for BGN and THBS2.
