# Supplementary material for: Identification of BGN and THBS2 as metastasis‐specific biomarkers and poor survival key regulators in human colon cancer by integrated analysis
Source: Clin Transl Med. 2022 Nov 14;12(11):e973. doi: 10.1002/ctm2.973 (PMC9663999; doi:10.1002/ctm2.973)
Supplement: Supplementary file 1 — Figure S1. The sample dendrogram and trait heatmap (A). Figure S2. The scatterplots of gene significance (GS) for histology vs. module membership (MM) in the black (A) and turquoise (B) modules. Figure S3. Correlation analysis revealed the significant positive correlation of 7 signature genes between the proteomic and transcriptomic profiles Figure S4. The overall and DFS survival of patients with different expression level of SPARC (A), CDH11 (B), MFAP2 (C), MMP11 (D), SPP1 (E) and THY1. [file CTM2-12-e973-s002.pdf]

Identification of BGN and THBS2 as metastasis-specific biomarkers and key regulators in human colon cancer by integrated analysis

Zhicheng He[1,2], Jian Lin[1,2], Cheng Chen[1,2], Yuanzhi Chen[1,2], Shuting Yang[1,3], Xianghai Cai[1], YingYing He[4, #], Shubai Liu[1, #]

[1] State Key Laboratory of Phytochemistry and Plant Resources in West China, Kunming Institute of Botany, Chinese Academy of Sciences, Kunming, 650201 Yunnan, China;

[2] University of Chinese Academy of Sciences, Beijing 100049, China;

[3] School of life Science, Yunnan University, Kunming, Yunnan 650091, China;

[4] School of Chemical Science & Technology, Yunnan University, Kunming, Yunnan 650091, China.

Correspondence to:

Dr. Shubai Liu, Mailing address: State Key Laboratory of Phytochemistry and Plant Resources in West China, Kunming Institute of Botany, Chinese Academy of Sciences, 132 Blue Black Street, Kunming, Yunnan 650201, China; Phone: (86) 871-65223309; E-mail: liushubai@mail.kib.ac.cn..

Or Dr. Yingying He, School of Chemical Science & Technology, Yunnan University, Kunming, Yunnan 650091, China, E-mail: Yingying.he10@gmail.com

.

Supplementary Materials and Methods:

Data processing and analysis

Briefly, the datasets contained about 22,483 target genes, which contains total of 45046 probes that correspond to measuring changes in gene expression profiles that are correlated with the physiologic profile of colon cancer tissues (Supplementary Table S1). Each gene expression value was log2-transformed and quantile normalized for transcriptome analysis using the limma package and scripts in R/Bioconductor [1]. According to the metastasis feature (Supplementary Table S1), biopsies expression profiles were divided to four subgroups, including normal colon tissues as control (n=69) and colon cancer tissues (without metastasis, n=322; with metastasis but no distal metastasis, n=212; with distal metastasis patients, n=78). Among all of the characteristics, data on metastasis, which is the subject of our research, stands out (Supplementary Table 2).

Construction of Weighted Gene Co-Expression Network and Identification Significance Module

The WGCNA package of R (version 1.63) was download and setup by following the protocol described previously [2]. The WGCNA package was used for performing various functions in weighted correlation network analysis, including constructing network, detecting module, calculating topological properties, simulating data, visualization, and interfacing with external software[2] . First of all, the quality of microarray was evaluated using sample clustering based on the distance between

different samples in Pearson's correlation matrices, and a height cut of 90 was chosen to identify potential microarray outliers. Four samples (GSM972311 / 972046 / 972213 / 972021) of colon cancer tissues were detected as outliers and ignored in the subsequent analysis. The colon cancer and health samples were separated in the PCA plot (Supplementary Figure. S1A), and the hierarchical clustering on the samples was performed to detect potential outliers. After data processed (**Figure S1A &B**), the whole transcription profiles of 677 patient's biopsy were used in this study, including the health (69 cases) and colon cancer patients (608 cases, Supplementary Table S1). The WGCNA and the hierarchical clustering were used to identify distinct modules related to colon cancer metastasis. The soft threshold $\beta = 7$ was chose to construct the co-expression network as the R^2 reached the peak for the first time when $\beta = 7$. The plot of log10(p(k)) versus log10(k) indicated that the network was close to a scale-free network by using $\beta = 7$, where k was the whole network connectivity and p(k) was the corresponding frequency distribution. When $\beta = 7$, the R^2 is 0.98, ensuring that the network was close to the scale-free network. After the soft thresholding power β was determined, the Topological Overlap Matrix (TOM) and dissTOM = 1−TOM were obtained. After the modules were identified, the T-test was used to calculate the significant p-value of candidate genes, and the gene significance (GS) was defined as mediated p-value of each gene (GS = lgP). Then, the module significance (MS) was defined as the average GS of all the genes involved in the module. The cut-off significant standard was setup as p-value lower than 0.05. In general, the module with the highest MS among all the selected modules will be considered as the one associated

with pathological feature metastasis. In the WGCNA, the module membership (MM): MM(i) = cor (xi, ME) is defined to measure the importance of the gene within the module. The greater absolute value of MM(i), the gene i is more important in the module. The Genes Significance (GS) in the module is highly correlated with MM and the most important element to discover the significant module, indicating that Genes in module is significantly associated with colon cancer metastasis feature. The hierarchical clustering analysis was used to identify gene modules and color to indicate modules, which is a cluster of densely interconnected genes in terms of co-expression. For genes that are not assigned to any of the modules, WGCNA places them in a grey module as not co-expressed. The module eigengene (ME) of a module is defined as the first principal component of the module and represents the overall expression level of the module. To identify modules that significantly associated with the traits of different etiologies, it was calculated the correlation of MEs (i.e. the first principle component of a module) [3] with clinical pathological features and identified the most significant associations. The correlation between different modules and the studied phenotype was calculated, the module with the strongest correlation with the studied disease characteristics were selected, and the correlation between modules was further verified by matrix and tree diagram.

Functions & Pathways analysis

The genes with the strongest significance and correlation were extracted from the significance modules and filtered in Genclip (http://ci.smu.edu.cn) to remove the duplicated genes. David database was used to enrich the signaling pathway, cell components and biological function of selected genes, and there are 111 genes corresponding to each significant enrichment were selected for further analysis.

Data analysis of signature gene expression profile

In order to further analysis hub genes' expression differences in non-metastatic and metastatic patients, the processed expression profile data (original data) were analyzed to detect the expression levels of eight hub genes in normal samples, cancer patients and metastatic cancer patients. Expression heat-map drawing by **MeV 4.9.0 (Multiple ExperimentViewer） and differences in expression level analysis by** GraphPad Prism 6.0 (GraphPad Software, La Jolla, CA, USA).

Survival analysis

To confirm the most significant signature associated to colon cancer metastasis. Eight hub genes' overall survival and disease-free survival were analyzed by GEPIA database. And two representative genes (BGN and THBS2) were screened from eight genes for further experimental verification after survival analysis.

Hematoxylin- Eosin (H&E) and Immunohistochemistry (IHC) staining

In order to detect the protein expression level of BGN and THBS2, colon cancer

tissue microarrays were purchased from Shanghai Outdo Biotech CO., LTD. (tissue samples from National Human Genetic Resources Sharing Service Platform, Serial number: 2005DKA21300). 4μm thick sections from the formalin-fixed, paraffin-embedded tissues. There are 160 colon tissues on the slide, it includes 80 tumor tissues and 80 adjacent tissues. Take the slide in a 60°C oven 1 hour, then deparaffinized in xylene and rehydrated through a graded series of ethanol solution. After the endogenous peroxidase was inhibited by 3% hydrogen peroxide for 10 min and the antigen was retrieved, the sections were incubated with antibody against BGN and THBS2 (Proteintech, ABclonal; 1:200) at 4°C overnight. Sections were then incubated with peroxidase (HRP)-conjugated secondary antibodies (BOSTER, China) according to the instructions of the BOSTER SABC detection protocol. For evaluation the difference of three genes' protein expression level in tumor and adjacent tissues, and positive staining rate was calculated as the expression level by Image J version 1.53.

Western blotting

Cells were cultured in six well plate, and washed twice with ice cold PBS (phosphate buffered saline), then 200uL cellular lysates (RIPA: PMSF, 100:1) were added in per well. The total protein concentration was determined by NanoDrop (Thermo Fisher Scientific, USA). Equal amounts (30~50ug) of whole cell lysates were separated on 8~12% SDS-PAGE gel, 80V for 30min, then 120V for well separated. And electrophoretically transferred onto polyvinylidene fluoride membranes (300mA, 120min~135min). Following blocking with 5% nonfat milk in PBST at room

temperature for 2h, then membranes were incubated with primary antibodies overnight at 4°C. The antibodies of BGN and THBS2 used to explore protein expression are same as description in "***Hematoxylin- Eosin (H&E) and Immunohistochemistry (IHC) staining***". Primary antibody of GAPDH (BBI CO., LTD. China). The secondary antibodies were incubated at 37°C for 2h, HRP-labeled goat anti-rabbit IgG (BBI CO., LTD. China) for three detection genes and HRP-labeled goat anti-mouse IgG for GAPDH (BBI CO., LTD. China). Finally, ECL luminescent solution was uniformly added to PVDF membranes, and the results were quantitatively analyzed by Image J after Tanon photography detection.

Cell Proliferation assay and colony formation assay

For CCK8 assay, 2,500 cells (100uL) per well were seeded into the 96-well plates and cultured for the indicated times. Then, 10uL of cell counting kit-8 (CCK8) solution (Proteintech, China) was added into each plate and incubated at 37°C incubator for 2h. The absorbance at 450 nm was measured by a microplate reader (BioTek Instruments, Winooski, VT, USA).

For colony formation assays, 500 cells per well were seeded into sixwell plates and cultured for 14 days. Then, clones were fixed and stained using 0.1% crystal violet. Colony contained more than 50 cells were counted under light microscopy (Leica DM Microscope, Germany). And the experiments were performed in triplicates.

Cell Migration and Invasion Assay

The ability of colon cancer cell migration and invasion were examined by transwell assays using 24-well transwell chambers with an 8-um pore size and a polycarbonate membrane (Corning, New York, NY, USA). In the migration assay, cells (5×10^4~1×10^5/well) were plated into the upper chamber of 8-mm-pore-size transwell chambers (Corning, New York, USA). Dulbecco's modified Eagle's medium containing 10% fetal bovine serum was added into the lower chamber. Then the chambers were incubated at 37°C for 24~48 h. Cells in the upper chamber were removed by Cotton swabs, and the cells on bottom surface of the membranes was fixed by 4% paraformaldehyde, then staining using 0.1% crystal violet dye. The remaining cells were photographed and counted using a photographic inverted microscope (Leica Microscope, Germany). In the invasion assay, Matrigel (Corning, New York, USA) was used in the transwell chambers. The other experimental procedures are the same as the migration assay.

Correlation analysis between signature gene expression pattern and clinical medication

The gene expression profiles data from ROC plotter (http://www.rocplot.org/crc/index), which is capable of linking gene expression and response to therapy using transcriptome-level data, were used to investigate the clinical prognosis of signature genes and the relationship between their expression levels and five clinical drug treatments for colon cancer. The correspond AUC of each gene and

drug also were examined. The influence for cell proliferation after 5-fluorouracil, irinotecan and oxaliplatin treated was measured by cell proliferation assay as shown in part "***Cell Proliferation assay and colony formation assay***".

Figure Cartoon

Figure 6G cartoon was designed by us and produced through Figdraw (https://www.figdraw.com) (our figure ID: USPTRdddde).

References:

1. Ritchie, M.E., et al., *limma powers differential expression analyses for RNA-sequencing and microarray studies.* Nucleic Acids Res, 2015. **43**(7): p. e47.
2. Langfelder, P. and S. Horvath, *WGCNA: an R package for weighted correlation network analysis.* BMC Bioinformatics, 2008. **9**: p. 559.
3. Li, A. and S. Horvath, *Network neighborhood analysis with the multi-node topological overlap measure.* Bioinformatics, 2007. **23**(2): p. 222-31.
4. Alley, M.C., et al., *Feasibility of drug screening with panels of human tumor cell lines using a microculture tetrazolium assay.* Cancer research, 1988. **48**(3): p. 589-601.
5. Varghese, F., et al., *IHC Profiler: an open source plugin for the quantitative evaluation and automated scoring of immunohistochemistry images of human tissue samples.* PLoS One, 2014. **9**(5): p. e96801.

Figure Legend

Supplementary Figure 1. The sample dendrogram and trait heatmap. (**A**). Sample clustering to detect outliers. (**B**). Scale independence and mean connectivity of data analysis. The distinct co-expression modules were identified that significantly related to different pathological features. (C). Clustering dendrogram of samples based on their Euclidean distance. The clinical feature traits were stage (T, N, M), meta (metastasis), overall survival (OS) and death**.** The white color means a low value, red means a high

value. (D) The correlation of Module-clinical traits. Each row corresponds to a module; each column corresponds to a clinical trait feature. Each cell contains the test statistic value and its corresponding p value from the linear mixed-effects model. Network of eigengene represents the relationships among the modules and the histological traits. There are twenty-one modules were detected through the dataset. Two modules were significantly positive correlated to metastasis feature of colon cancer, including red (t-value = 0.16, p-value = 3e-05), black (t-value = 0.15, p-value = 6e-05).

Figure S2. The scatterplots of Gene Significance (GS) for histology vs. Module Membership (MM) in the black (A) and turquoise (B) modules. There is a highly significant correlation between GS and MM in this module, implying that the most important (central) elements of modules also tend to be highly correlated with cancer metastasis pathological trait, including black (Cor = 0.29, p-value = 4.3e-41), turquoise (t-value = 0.34, p-value = 2.1e-173).

Figure S3. Correlation analysis revealed the significant positive correlation of 7 signature genes between the proteomic and transcriptomic profiles. It was compared between of in no-metastasis and metastasis samples, respectively.

Figure S4. The overall and DFS survival of patients with different expression level of SPARC (A), CDH11(B), MFAP2 (C), MMP11(D), SPP1 (E) and THY1(F).

Supplementary Table S1. The biopsy samples information of gene expression profile.

Supplementary Table S2. All genes in red module.

Supplementary Table S3. The significant genes related to colorectal cancer metastasis.

Supplementary Table S4. 111 significant genes filtered by DAVID Enrichment

Supplementary Table S5. Top 250 genes up-regulated in COAD by GEPIA analysis

Supplementary Table S6. Transcriptomic profiles of 8 signature genes in CAPTC

Supplementary Table S7. Proteomics profiles of 7 signature genes

Supplementary Table S8. UMS proteomic profiles of 7 signature genes.

Supplementary Table S9. EMT proteomic profiles of 7 signature genes.

Supplementary Table S10. CMS1-4 proteomic profiles of 7 signature genes.

Supplementary Table S11. 5 clinical drug treatment response with or without BGN.

Supplementary Table S12. 5 clinical drug treatment response with or without THBS2.

Supplementary Table S13. shrna constructions for BGN and THBS2.

Figure S1.

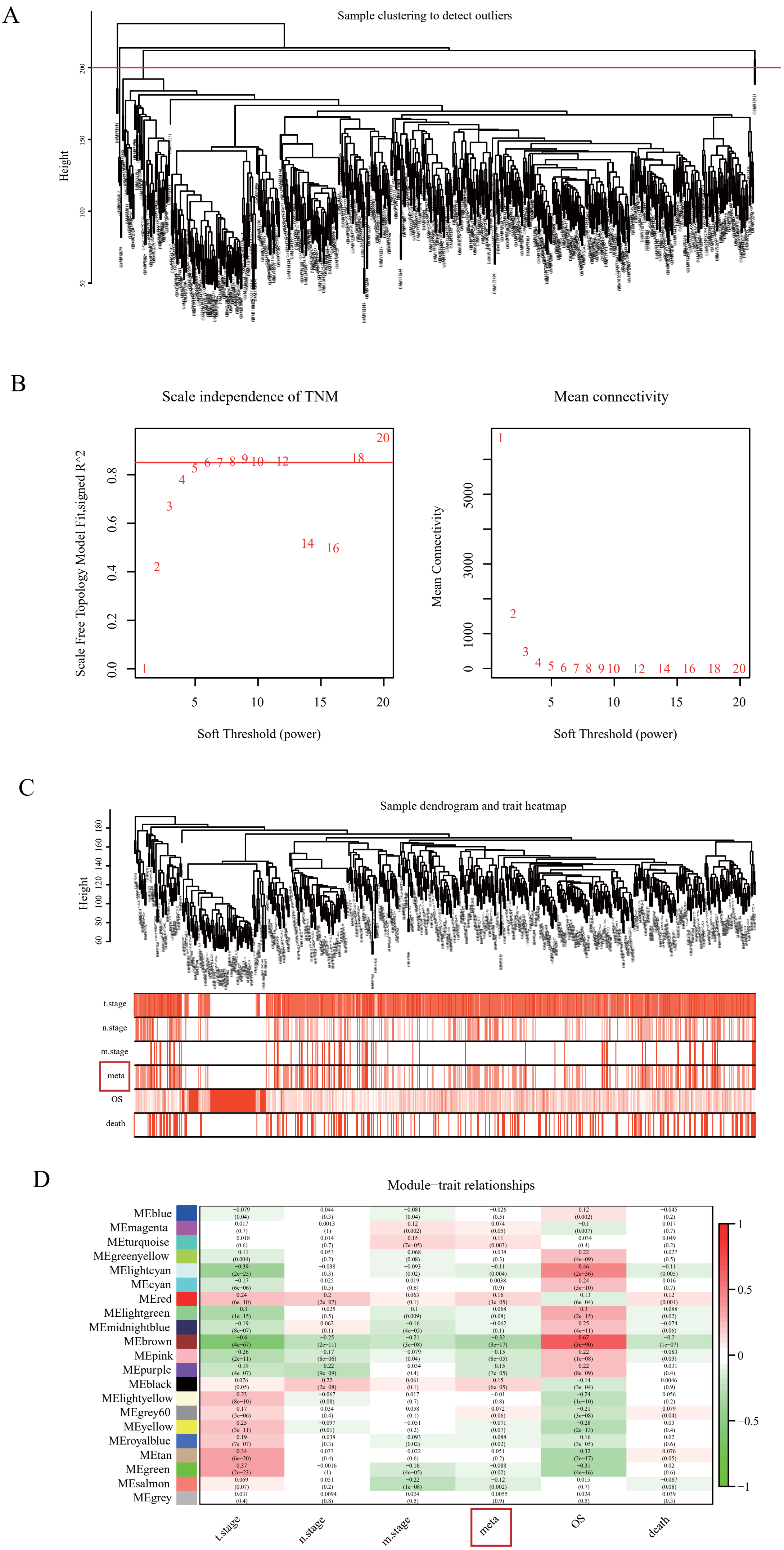
A
Sample clustering to detect outliers
Height
B
Scale independence of TNM
Mean connectivity
Scale Free Topology Model Fit,signed R^2
Mean Connectivity
Soft Threshold (power)
Soft Threshold (power)
C
Sample dendrogram and trait heatmap
Height
t.stage
n.stage
m.stage
meta
OS
death
D
Module−trait relationships
MEblue
MEmagenta
MEturquoise
MEgreenyellow
MElightcyan
MEcyan
MEred
MElightgreen
MEmidnightblue
MEbrown
MEpink
MEpurple
MEblack
MElightyellow
MEgrey60
MEyellow
MEroyalblue
MEtan
MEgreen
MEsalmon
MEgrey
t.stage
n.stage
m.stage
meta
OS
death

Figure S2.

A

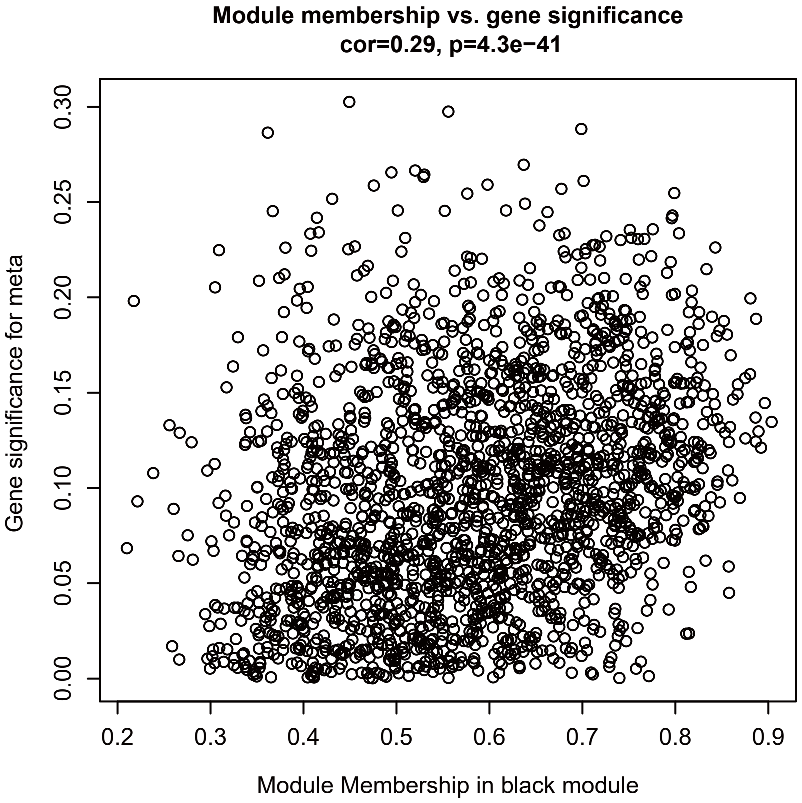
Module membership vs. gene significance
cor=0.29, p=4.3e−41
Gene significance for meta
0.00
0.05
0.10
0.15
0.20
0.25
0.30
0.2
0.3
0.4
0.5
0.6
0.7
0.8
0.9
Module Membership in black module

B

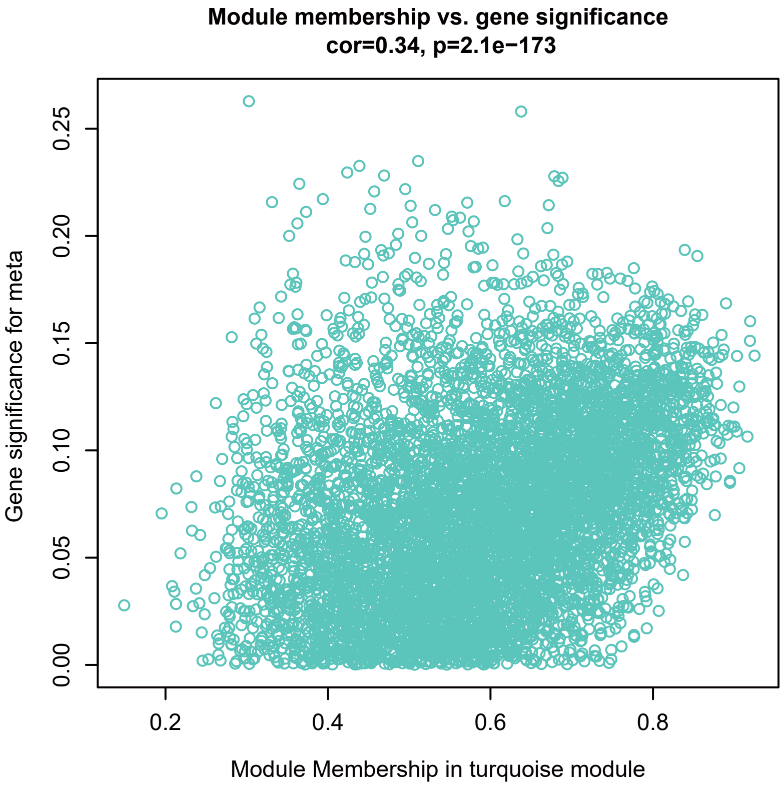
Module membership vs. gene significance
cor=0.34, p=2.1e−173
Gene significance for meta
0.00
0.05
0.10
0.15
0.20
0.25
0.2
0.4
0.6
0.8
Module Membership in turquoise module

Figure S3

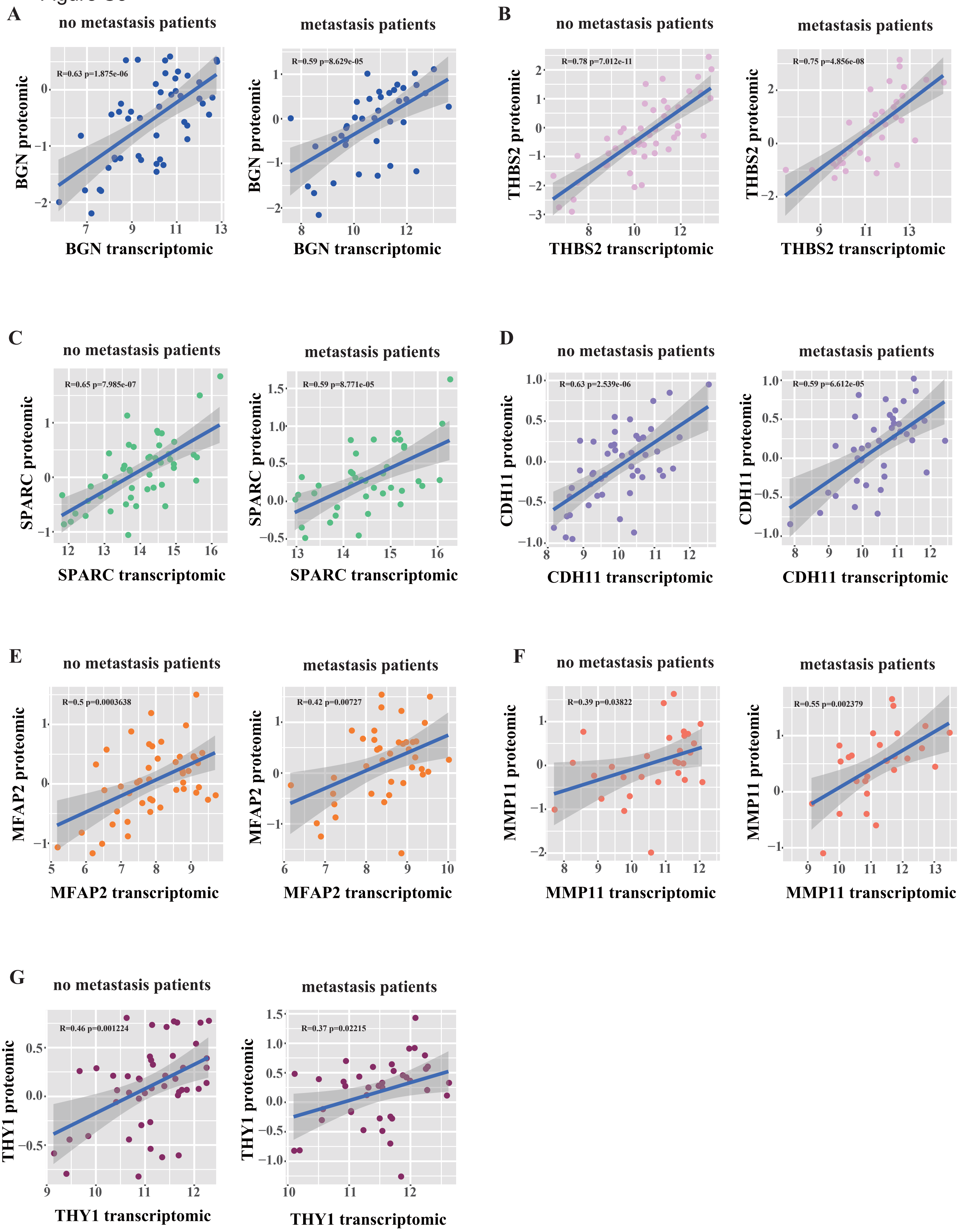
A
no metastasis patients
R=0.63 p=1.875e-06
BGN proteomic
BGN transcriptomic
metastasis patients
R=0.59 p=8.629e-05
BGN proteomic
BGN transcriptomic
B
no metastasis patients
R=0.78 p=7.012e-11
THBS2 proteomic
THBS2 transcriptomic
metastasis patients
R=0.75 p=4.856e-08
THBS2 proteomic
THBS2 transcriptomic
C
no metastasis patients
R=0.65 p=7.985e-07
SPARC proteomic
SPARC transcriptomic
metastasis patients
R=0.59 p=8.771e-05
SPARC proteomic
SPARC transcriptomic
D
no metastasis patients
R=0.63 p=2.539e-06
CDH11 proteomic
CDH11 transcriptomic
metastasis patients
R=0.59 p=6.612e-05
CDH11 proteomic
CDH11 transcriptomic
E
no metastasis patients
R=0.5 p=0.0003638
MFAP2 proteomic
MFAP2 transcriptomic
metastasis patients
R=0.42 p=0.00727
MFAP2 proteomic
MFAP2 transcriptomic
F
no metastasis patients
R=0.39 p=0.03822
MMP11 proteomic
MMP11 transcriptomic
metastasis patients
R=0.55 p=0.002379
MMP11 proteomic
MMP11 transcriptomic
G
no metastasis patients
R=0.46 p=0.001224
THY1 proteomic
THY1 transcriptomic
metastasis patients
R=0.37 p=0.02215
THY1 proteomic
THY1 transcriptomic

Figure S4

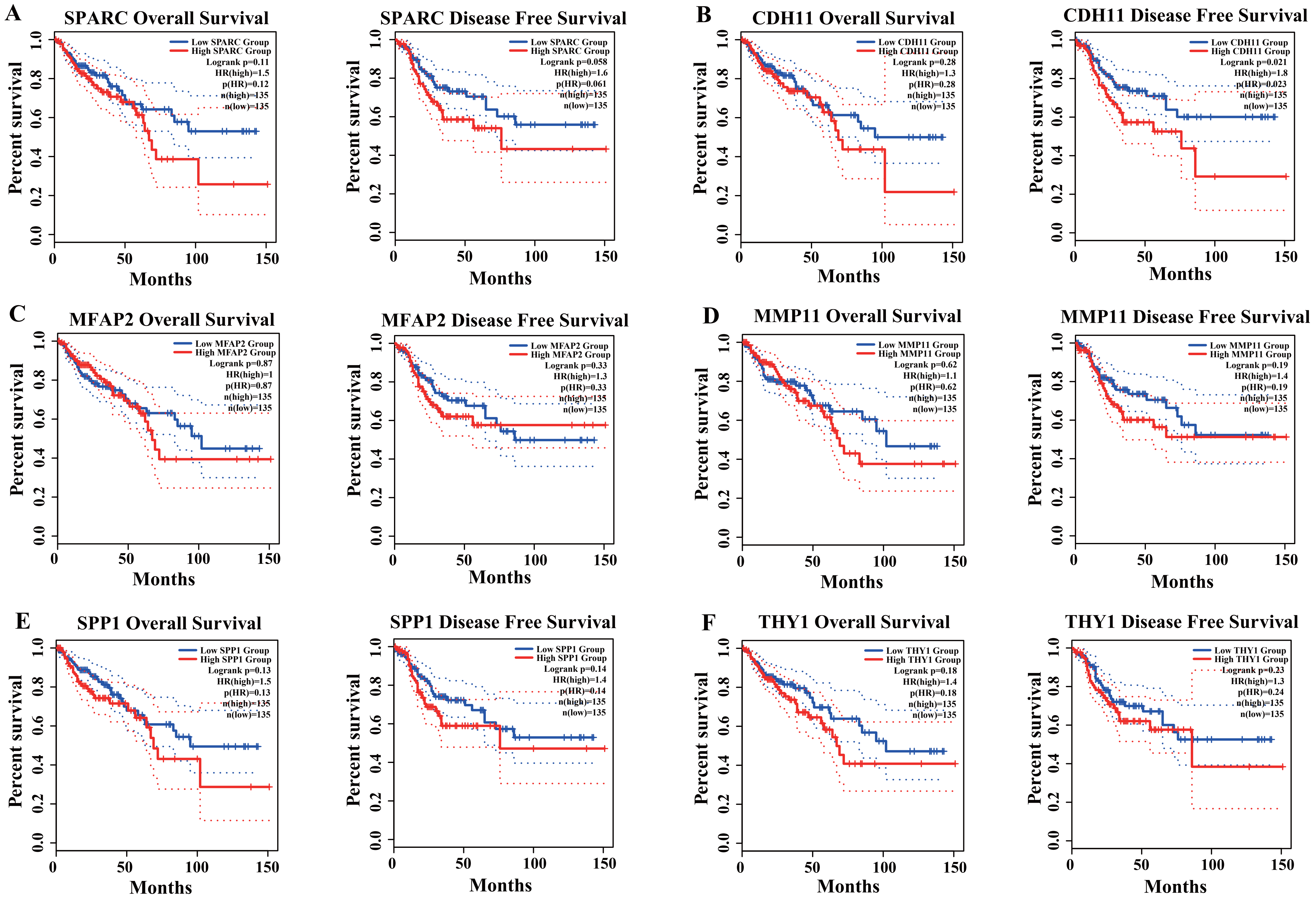
A
SPARC Overall Survival
Low SPARC Group
High SPARC Group
Logrank p=0.11
HR(high)=1.5
p(HR)=0.12
n(high)=135
n(low)=135
Percent survival
Months
SPARC Disease Free Survival
Low SPARC Group
High SPARC Group
Logrank p=0.058
HR(high)=1.6
p(HR)=0.061
n(high)=135
n(low)=135
Percent survival
Months
B
CDH11 Overall Survival
Low CDH11 Group
High CDH11 Group
Logrank p=0.28
HR(high)=1.3
p(HR)=0.28
n(high)=135
n(low)=135
Percent survival
Months
CDH11 Disease Free Survival
Low CDH11 Group
High CDH11 Group
Logrank p=0.021
HR(high)=1.8
p(HR)=0.023
n(high)=135
n(low)=135
Percent survival
Months
C
MFAP2 Overall Survival
Low MFAP2 Group
High MFAP2 Group
Logrank p=0.87
HR(high)=1
p(HR)=0.87
n(high)=135
n(low)=135
Percent survival
Months
MFAP2 Disease Free Survival
Low MFAP2 Group
High MFAP2 Group
Logrank p=0.33
HR(high)=1.3
p(HR)=0.33
n(high)=135
n(low)=135
Percent survival
Months
D
MMP11 Overall Survival
Low MMP11 Group
High MMP11 Group
Logrank p=0.62
HR(high)=1.1
p(HR)=0.62
n(high)=135
n(low)=135
Percent survival
Months
MMP11 Disease Free Survival
Low MMP11 Group
High MMP11 Group
Logrank p=0.19
HR(high)=1.4
p(HR)=0.19
n(high)=135
n(low)=135
Percent survival
Months
E
SPP1 Overall Survival
Low SPP1 Group
High SPP1 Group
Logrank p=0.13
HR(high)=1.5
p(HR)=0.13
n(high)=135
n(low)=135
Percent survival
Months
SPP1 Disease Free Survival
Low SPP1 Group
High SPP1 Group
Logrank p=0.14
HR(high)=1.4
p(HR)=0.14
n(high)=135
n(low)=135
Percent survival
Months
F
THY1 Overall Survival
Low THY1 Group
High THY1 Group
Logrank p=0.18
HR(high)=1.4
p(HR)=0.18
n(high)=135
n(low)=135
Percent survival
Months
THY1 Disease Free Survival
Low THY1 Group
High THY1 Group
Logrank p=0.23
HR(high)=1.3
p(HR)=0.24
n(high)=135
n(low)=135
Percent survival
Months
